# Solitary Confinement and Insanity

**Published:** 1897-08-14

**Authors:** 


### Modern Sociology.

### SOLITARY CONFINEMENT AND INSANITY.

There has always been a widespread feeling among mankind that complete isolation from his fellows tends to affect unfavourably a man's disposition, and even to upset his mental balance. From early times, next to the infliction of direct bodily torture, the depriving a man of personal liberty and of intercourse with his fellows has been the chief mode of his punishment for crime. Under our old prison system all criminals and suspects were huddled together in a filthy common room, just as one sees them in Tangiers to-day. The moment thoughtful and benevolent men began seriously to consider the question of the proper way to deal with the criminal the manifold evils of this system were seen, and the legislatures of all civilised countries became convinced that it must be changed. Apart from theoretical views of the nature of punishment, it became clear to men of common sense that the criminal should be reformed, as well as punished, if possible. The "separate system" of a cell for each prisoner was devised, and during the second half of this century a large sum of money was spent in reconstructing all the prisons of the Empire on this principle. The "silent system" of association, but with no speech allowed, was tried and failed. At first the separation was very stringent, and there is reason to believe that in some cases insanity was brought on in consequence. As usual, the sentimentalists and the professional purveyors of sensation exaggerated and distorted the facts. The instincts of human nature as to the terrible effects of solitary confinement tended towards the ready acceptance of the stories of madness and suicide being rampant under the solitary system. Of late we have heard little of these stories, and we may conclude that it is working well. In America they have tried many systems, to the enormous advantage of our scientific knowledge of "Penology." In Belgium a distinct advance has been made in that scientific knowledge by the recent appointment of Dr. Morel, a physician skilled in mental diseases, as official inspector and examiner of prisoners in regard to their mental condition and the effects of confinement on their minds. He gives the result of his experience in a very interesting and valuable paper on "Insanity and Solitary Confinement," written at the suggestion of the American Howard Association. Instead of vague impressions, we have there facts and statistics compiled by a competent expert in medicine, showing conclusively that prisoners may receive the full benefits of isolation from evil influences, and yet undergo sufficient punishment, while they are granted enough satisfaction to their natural social instincts to keep them from becoming insane. "When cellular imprisonment becomes absolute solitude it is, if unduly prolonged, an unwarrantable cruelty. Solitude is one thing, wise separation another. Continuous isolation is unnatural and ruinous to mind and body, whereas separation from evil associations only is most beneficial to its subjects." "The prisoner does not remain the whole day in his cell; once or twice a day he leaves it to go to his yard—each prisoner has a special yard to walk in—and during that time he is allowed to smoke." "He goes to chapel, to school, to the offices of the superintendent and the trades instructor." His time in the cell is interrupted by visits from the superintendent, the doctors, the chaplain, the schoolmaster, the workmaster, his guardians, members of the After-care Association, and the inspectors. Conversation urging moral conduct is allowed to every visitor. Each prisoner has in his cell five or six books, which are changed periodically. He is allowed to write and see his relations at times; he can himself deposit letters in a special box, addressed to the Board of Supervision; he is seldom idle, and his day is broken up as described. Dr. Morel visits the prisons regularly, and carefully examines every prisoner who shows any sign of mental disturbance. In that way the most merciful and truly scientific object is attained of early symptoms of insanity being seen and treated at once, and of weak and pre-disposed brains being specially dealt with. In this way frequent cases where crime is really an early symptom of commencing insanity are detected, and the patients at once placed under medical treatment. All the best-disposed of the prisoners like this system and are benefited by it, while there is no reason whatever to think that more of them become insane than if they were at large. On the contrary, the system tends towards sanity, for it increases the faculty of mental inhibition, and it is more and more apparent as a fact in medico-psychology that self-control makes for sanity—is sanity—and the lack of it is insanity.

Dr. Morel is gradually building up a trustworthy mass of unbiassed facts on a most important point—the real relationship of crime to mental defect. We shall by and bye, by means of such facts, be able to judge between the schools of Lombroso and of the North. Morel makes the medico-psychological examination of each mentally-suspected prisoner according to a most complete and uniform system, examining into bodily and mental heredity, into the diseases of childhood, the education, the employment, the associations, and the temptations to which the prisoner has been subject. In addition, he makes a careful psychological and an exhaustive physical examination of the case. He says: "By such means, I doubt not, crime and punishment will be studied in a future time in a new way, and with this result—that the population of the prisons will diminish, and that the very numerous young offenders between 18 and 25 to 30 years of age, being really degenerate and uneducated, will be taken to special institutions, where, instead of being imprisoned for a few months or a few years, they will receive a special education till another medico-psychological examination declares them to bear the name of a good citizen." This is a truly scientific way of attacking a tremendous problem in which the human brain plays the chief part. We trust that in Great Britain we may in due time follow on the same lines. We have done much in this country to solve the problem of the right treatment of the criminal and the prevention of crime, but we have trusted hitherto to unaided common sense and philanthropic feeling, rather despising science and its teachings. We believe that the science of medicine and the objective study of psychology will be able in the future greatly to aid the legislator and the prison administrator in this important matter.

